# Clinical characteristics of coronavirus disease 2019 (COVID-19) in patients out of Wuhan from China: a case control study

**DOI:** 10.1186/s12879-021-05897-z

**Published:** 2021-02-24

**Authors:** Hua Zhang, Feng Du, Xiao-jun Cao, Xia-long Feng, He-ping Zhang, Zheng-xia Wu, Bao-Feng Wang, Hong-juan Zhang, Rui Liu, Jian-jun Yang, Bo Ning, Kai Chen, Zhen-peng Huang

**Affiliations:** 1 Present Address: Department of Pulmonary and Critical Care Medicine, Baoji Central Hospital, No. 8 Jiangtan Rd, Baoji, 721008 Shaanxi China; 2 Department of Infectious Diseases, Baoji Central Hospital, Baoji, 721008 Shaanxi China; 3 Department of Cardiovascular Medicine, Baoji Central Hospital, Baoji, 721008 Shaanxi China; 4 Present Address: Guangzhou Institute of Oncology, Affiliated Cancer Hospital & Institute of Guangzhou Medical University, No. 78 Hengzhigang, Guangzhou, 510095 Guangdong China

**Keywords:** 2019-nCoV, COVID-19, Clinical characteristics, Wuhan, China

## Abstract

**Background:**

A large-scale global outbreak of coronavirus disease-19 (COVID-19) out of Wuhan, from China, occurred in January 2020. To examine the clinical characteristics of COVID-19 in infected patients out of Wuhan, from China.

**Methods:**

Thirteen patients were confirmed to be infected with novel coronavirus-2019 (2019-nCoV) between January 27 and February 8, 2020, in Baoji city, Shannxi, northwestern China. Epidemiological and clinical information, and computed to morphology imaging data from all COVID-19 patients were collected; cases were divided into two groups according to the severity of infection (mild or severe).

**Results:**

Nine (9/13) COVID-19 patients exhibited mild disease severity, and defined as second-generation human-to-human transmission cases. Most patients (11/13) had a history of travel to or from Wuhan. There were no differences in sex and age between the mild and severe cases (all *P* > 0.05). A moderate degree of fever (11/13), cough (13/13), and fatigue (8/13) were common symptoms; however, there was no statistical difference between mild and severe cases in this regard (all *P* > 0.05). Oxyhemoglobin saturation and oxygenation index decreased, and C-reactive protein (CRP) and serum amyloid A (SAA) levels were elevated in all patients with COVID-19 infection, with statistically significant differences between those with severe disease and mild infection (all *P* < 0.05). Twelve of 13 COVID-19 patients exhibited changes in chest CT imaging features, and time course changes were different between mild and severe cases (all *P* < 0.05).

**Conclusion:**

Most cases of COVID-19 infection were second-generation human-to-human transmissions from Wuhan and were mild in severity. The clinical characteristics of COVID-19 varied. Oxyhemoglobin saturation, oxygenation index, CRP and SAA levels, and CT features were reliable parameters to evaluate the severity of COVID-19 infection. However, a few patients with mild COVID-19 disease lacked typical characteristics such as fever and changes in CT imaging features.

## Background

A few cases of pneumonia associated with exposure to the Wuhan Seafood market (Wuhan, China) were reported and found to be caused by novel coronavirus-2019 (2019-nCoV) in December 2019 [1, 2]. The 2019-nCoV infection emerged on a large scale from Wuhan and spread all over the world in January 2020, and is now known to transmitted by person-to-person contact [3]. The World Health Organization (WHO) declared the outbreak of 2019-nCoV to be a public health emergency of international concern on January 30, 2020. The novel coronavirus was of lineage B of the genus beta-coronavirus of the coronavirus family, of which severe acute respiratory syndrome-related coronavirus (SARS-CoV) and Middle East respiratory syndrome-related coronavirus (MERS-CoV) are also included [4]. The International Committee on Taxonomy of Viruses defined it as SARS-CoV-2 on February 11, 2020. The WHO also defined “coronavirus disease 2019” (COVID-19) in patients infected with 2019-nCoV on the same day. SARS-CoV-2 is the third most fatal virus in the coronavirus family, which is weaker than MERS-CoV (37% fatality rate) and SARS-CoV (10% fatality rate) [5, 6]. A recent study reported that fever, cough, and fatigue are common symptoms among patients infected with COVID-19 [1]. However, the clinical characteristics of patients with COVID-19 remain unclear. Moreover, whether the clinical characteristics of COVID-19 are different from those of Wuhan and other regions of China has not yet been reported.

Accordingly, the present study aimed to examine the clinical characteristics of COVID-19 infection in patients out of Wuhan, Hubei, from China. We collected and analyzed data from COVID-19 cases in Baoji city, Shaanxi, northwestern China.

## Methods

### Patients

Thirteen patients admitted to Baoji Central Hospital were confirmed to be infected with 2019-nCoV using real-time polymerase chain reaction (PCR) technique sat the Baoji City Center for Disease Control and Prevention (CDC) between January 27 and February 8, 2020. Initial onset of disease symptoms occurred from January 18 to February 4, 2020.

### Examinations

Clinical biomedical information, including routine blood work-up, C-reactive protein (CRP), procalcitonin (PCT) and serum amyloid protein A (SAA), and computed tomography (CT) imaging of all COVID-19 patients with the CDC laboratory-confirmed infection with 2019-nCoV were collected at the earliest possible time.

According to the China precept on diagnosis and treatment of novel coronavirus pneeumoia (Revised criteria, fifth edition), COVID-19 patients with severe respiratory distress (respiratory rate ≥ 30 breaths/min), hypoxemia oxyhemoglobin saturation ≤ 93%, partial pressure of arterial oxygen (PaO_2_)/oxygen saturation (SpO_2_) ≤ 300 mmHg, respiratory failure, shock, and multiple organ dysfunction syndrome was defined as severe COVID-19 infection [7].

The present study was approved by the Institutional Ethical Committee of Baoji Central Hospital. Verbal and written informed consents were obtained from all COVID-19 patients and/or their family members.

### Statistical analysis

Statistical analysis was performed using SPSS version 25.0 (IBM Corporation, Armonk, NY, USA) for Windows (Microsoft Corporation, Redmond, WA, USA). Continuous variables are expressed as mean ± standard deviation (SD) and were compared using the Student’s t test. Categorical variables are presented as proportions and percentages. Categorical variables were compared using the chi-squared test and rank sum test, respectively. Differences with *P* < 0.05 (two-sided) was considered to be statistically significant.

## Results

### Epidemiological characteristics of COVID-19 patients

A total of 13 patients with COVID-19 infection (7 male and 6 female; sex ratio 1:1.17; mean age [± SD] age, 49.54 ± 10.85 years [range 27–71 years]) were examined. Most patients were employees or farmers, and had a history of travel to or from Wuhan, or other cities in Hubei province within the preceding 2 weeks. Patients out of Wuhan or Hubei province, most COVID-19 cases were second-generation human-to-human transmissions. Disease duration ranged from 3 to 11 days, mean 6.23 ± 2.42 days (initial onset to confirmation of infection with 2019-nCoV). Most patients, who were from Wuhan or Hubei, were diagnosed with mild COVID-19 infection (Table 1).

**Table 1:** Epidemiological characteristics of COVID-19 patients

Epidemiological Characteristics	
**Sex**	
Male	7 (53.8%)
Female	6 (46.2%)
**Age (Years)**	27–71 (49.54 ± 10.85)
**Occupation**	
Employee	6 (46.2%)
Farmer	5 (38.4%)
Teacher	1 (7.7%)
Unemployed	1 (7.7%)
**History of Travel to or From Wuhan, Hubei**	
Yes	11 (84.62%)
No	2 (15.38%)
**Patients’ Generation of Human-to-human Transmission**	
First Generation	2 (15.38%)
Second Generation	9 (69.24%)
Third Generation	2 (15.38%)
**From Initial Onset to Confirm Infected** 2019-nCoV **(Day)**	3–11 (6.23 ± 2.42)
**Severity of** COVID-19	
Mild	9 (69.24%)
Severe	4 (30.76%)

In those with mild COVID-19 infection, there was no difference in sex; most severe COVID-19 cases were male; however, this difference was not statistically significant (*P* > 0.05) (Table 2). There were also no differences in age and disease duration between the mild and severe COVID-19 cases (all *P* > 0.05) (Table 3).

**Table 2:** Gender characteristics of different severity of COVID-19

Sex #	Male	Female
**Mild** Cases **(*n*** **= 9)**	4 (44.44%)	5 (55.56%)
**Severe** Cases **(*n*** **= 4)**	3 (75%)	1 (25%)

# *x*^2^ = 0.1741, *P* > 0.05

**Table 3:** Ages and diagnosis time in different severity of COVID-19 cases

Ages and Diagnosis Time	
**Age (Year)**	
Mild Cases	27–71 (47.00 ± 11.74)
Severe Cases	50–64 (55.25 ± 6.40)
	*t* = −1.301, *P* > 0.05
**From Initial Onset to Confirm Infected** 2019-nCoV **(Day)**	
Mild Cases	3–11 (6.67 ± 2.69)
Severe Cases	4–7 (5.25 ± 1.50)
	*t* = 0.972, *P* > 0.05

### Characteristics of clinical symptoms in COVID-19 patients

All COVID-19 patients exhibited symptoms of cough, and most had a moderate degree of fever (38.0–39.0 °C); however, two did not exhibit fever. Most of those infected with COVID-19 and exhibited fever varying from 1 to 9 days (mean 3.09 ± 3.21 days), and persisting 4 to 11 days (mean 7.55 ± 3.08 days). Fever was not the only screening criterion for COVID-19 infection. Almost one-half of COVID-19 patients exhibited expectoration, fatigue, and gastrointestinal symptoms such as anorexia, nausea, vomiting, and diarrhea. One of the COVID-19 patients experienced dyspnea and pharyngalgia. Most patients’ oxyhemoglobin saturation (90–98% [mean 95.77 ± 2.74%) and oxygenation index (203–462 mmHg [mean 324.11 ± 96.20 mmHg]) were decreased in the early period of 2019-nCoV infection.

More than one-half of patients infected by COVID-19 had one to three concomitant diseases, such as hypertension, diabetes, and/or cerebral infarction. One patient exhibited acute mental disorder; as such, devoting attention both physiological and mental disorders in COVID-19 patients is important (Table 4).

**Table 4:** Clinical symptoms and concomitant diseases in COVID-19 patients

Clinical Symptoms and Concomitant Diseases	
**Fever**	
Yes	11 (84.62%)
No	2 (15.38%)
**From Initial Onset to to Fever (Day)**	1–9 (3.09 ± 3.21)
**Degree of Fever (°C)**	38.0–39.0 (38.35 ± 0.40)
**Day of Persistent Fever**	4–11 (7.55 ± 3.08)
**Cough**	13 (100%)
**Expectoration**	5 (38.46%)
**Fatigue**	8 (61.54%)
**Gastrointestinal Symptoms**	6 (46.15%)
**Dyspnea**	1 (7.69%)
**Pharyngalgia**	1 (7.69%)
**Oxyhemoglobin Saturation (%)**	90–98 (95.77 ± 2.74)
**Oxygenation Index (mmHg)**	203–462 (324.11 ± 96.20)
**Concomitant Diseases**	
Yes	8 (61.54%)
No	5 (38.46%)
**Numbers of Concomitant Diseases**	
1	3 (37.5%)
2	3 (37.5%)
3	2 (25%)

### Characteristics of clinical symptoms and severity of COVID-19 patients

Cough and fever were common clinical manifestations of COVID-19. There were no differences between mild and severe cases; however, a few patients with mild COVID-19 disease did not exhibit fever. Severe COVID-19 cases were more likely to experience expectoration and fatigue; however, the difference was not statistically significant (all *P* > 0.05). Oxyhemoglobin saturation and oxygenation were both sensitive indices for identification of mild and severe COVID-19 (all *P* < 0.05). Patients with severe COVID-19 were significantly more likely to exhibit hypoxia and respiratory distress (Table 5).

**Table 5:** Clinical symptoms in different severity of COVID-19 cases

Clinical Symptoms	
**Fever**	
Mild Cases	7 (77.78%)
Severe Cases	4 (100%)
	*x*^2^ = 0.0369, *P* > 0.05
**From Initial Onset to Fever (Day)**	
Mild Cases	1–9 (3.29 ± 3.59)
Severe Cases	1–7 (2.75 ± 2.87)
	*t* = 0.254, *P* > 0.05
**Degree of Fever (°C)**	
Mild Cases	36.5–39.0 (37.91 ± 0.86)
Severe Cases	38.0–39.0 (38.40 ± 0.49)
	*t* = −1.043, *P* > 0.05
**Day of Persistent Fever**	
Mild Cases	4–11 (6.13 ± 4.70)
Severe Cases	5–10 (7.50 ± 2.89)
	*t* = −0.624, *P* > 0.05
**Cough**	
Mild Cases	9 (100%)
Severe Cases	4 (100%)
**Expectoration**	
Mild Cases	3 (33.33%)
Severe Cases	2 (50%)
	*x*^2^ = 0.0021, *P* > 0.05
**Fatigue**	
Mild Cases	4 (44.44%)
Severe Cases	4 (100%)
	*x*^2^ = 3.61, *P* > 0.05
**Other Symptoms**	
Mild Cases	5 (55.56%)
Severe Cases	3 (75%)
	*x*^2^ = 0.4424, *P* > 0.05
**Oxyhemoglobin Saturation (%)**	
Mild Cases	95–98 (97.00 ± 1.80)
Severe Cases	90–94 (93.00 ± 2.58)
	*t* = 3.255, *P* < 0.05
**Oxygenation Index (mmHg)**	
Mild Cases	342–462 (398.60 ± 50.91)
Severe Cases	203–252 (231.00 ± 20.41)
	*t* = 6.133, *P* < 0.05

The present study also confirmed that patients with concomitant diseases were more likely to deteriorate in the early stages of COVID-19 infection (*P* < 0.05) (Table 6).

**Table 6:** Concomitant diseases in different severity of COVID-19 cases

Numbers of Concomitant Diseases	0	1	2	3
**Mild** COVID-19 **(*n*** **= 9)**	5 (55.56%)	3 (33.33%)	1 (11.11%)	0
**Severe** COVID-19 **(*n*** **= 4)**	0	0	2 (50%)	2 (50%)

Numbers of concomitant diseases and severity of COVID-19 infection # *x*^2^ = 11.0963, *P* < 0.05

### Clinical biomedical characteristics of COVID-19 patients

In the early period of COVID-19 infection, routine blood work-up, including white blood cells, neutrophils, lymphocytes and monocytes, did not change significantly, and white blood cells and neutrophils were higher in severe cases than in mild cases (all *P* < 0.05).

PCT levels did not increase in the early period of COVID-19 infection, and there was no difference between those with mild and severe disease. In contrast, CRP and SAA levels increased rapidly in all COVID-19 cases, and the levels of these two biomarkers were significantly higher in those with severe disease than in mild cases (all *P* < 0.05). As such, CRP and SAA may be sensitive indices for detecting COVID-19 and classifying disease severity (Table 7).

**Table 7:** Blood biomedical characteristics of COVID-19 patients

	Total Cases	Mild Cases*	Severe Cases*	
**White Blood Cell (× 10**^**9**^**/L)**	3.97 ± 0.89	3.58 ± 0.70	4.85 ± 0.60	* t = −3.132, *P* < 0.05
**Neutrophil (× 10**^**9**^**/L)**	2.46 ± 0.76	2.12 ± 0.56	3.25 ± 0.52	* t = − 3.410, *P* < 0.05
**Lymphocyte (×10**^**9**^**/L)**	0.98 ± 0.21	0.99 ± 0.25	0.95 ± 0.09	* t = 0.368, *P* > 0.05
**Monocyte (× 10**^**9**^**/L)**	0.48 ± 0.25	0.41 ± 0.18	0.63 ± 0.34	* t = −1.586, *P* > 0.05
**CRP (mg/L)**	20.07 ± 24.66	9.84 ± 11.90	43.09 ± 32.17	* t = −2.819, *P* < 0.05
**PCT (ng/L)**	0.04 ± 0.03	0.03 ± 0.02	0.05 ± 0.03	* t = −0.11, *P* > 0.05
**SAA (mg/L)**	93.00 ± 111.17	36.41 ± 47.18	268.93 ± 33.13	* t = −8.835, *P* < 0.05

*Blood biomedical levels between mild COVID-19 cases and severe COVID-19 cases

### CT imaging changes in COVID-19 patients

Almost every COVID-19 patient exhibited imaging changes on chest CT scans, with bilateral lung lesions in severe cases, with both unilateral and bilateral lung lesions in patients with mild infection. However, one patient with mild disease did not exhibit significant lung lesions (*u-* = 3.1542, *P* < 0.05).

**Fig. 1:**
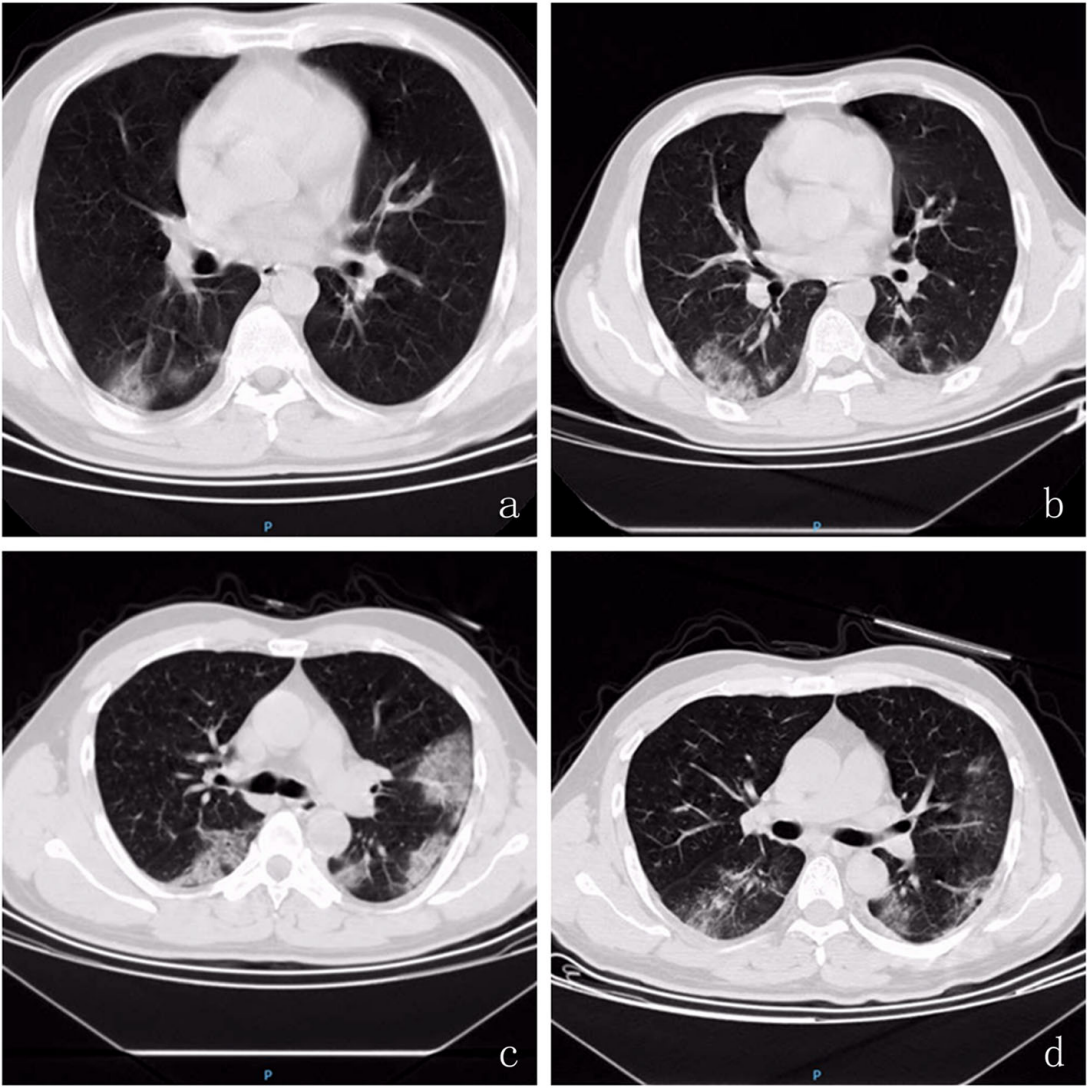
Chest CT scan image changes in COVID-19 Patients. At the day of confirm infection of 2019-nCoV, chest CT showed that multiple patchy sub-segmental or segmental ground-glass density shadow and consolidation were in bilateral lung (**a** severe cases & **c** mild cases); **b** Severe COVID-19 patient 5 days after confirm infection of 2019-nCoV, ground-glass density shadow and consolidation in bilateral lung was exacerbation; **d** Mild COVID-19 patient 5 days after confirm infection of 2019-nCoV, ground-glass density shadows in bilateral lung was absorbed

Chest CT scan image changes, such as multiple patchy sub-segmental or segmental ground-glass opacities, shadows or consolidations in the bilateral lung, were found among COVID-19 patients. Five days after confirmation of infection with 2019-nCoV, CT imaging revealed that shadows or consolidations were absorbed in the lungs in more than one-half of mild cases; however, this was exacerbated in most severe cases (*u-* = 1.9748, *P* < 0.05). Chest CT scan was an effective method to examine and evaluate the severity of COVID-19 infection (Table 8 and Fig. 1).

**Table 8:** CT imaging changes in COVID-19 patients

	Mild Cases (***n*** = 9)	Severe Cases (***n*** = 4)
**Location of Lung lesion**		
**No Lung Lesion**	1 (11.11%)	0
**Unilateral Lung Lesion**	1 (11.11%)	0
**Bilateral Lung Lesion**	7 (77.78%)	4 (100%)
	*u-* = 3.1542, *P* < 0.05	
**Day 5 After Confirmed Infection of 2019-nCoV CT Imaging Change**		
**Exacerbation**	2 (22.22%)	3 (75%)
**No change**	2 (22.22%)	0
**Absorption**	5 (55.56%)	1 (25%)
	*u-* = 1.9748, *P* < 0.05

## Discussion

In the present study, 13 individuals were confirmed to be infected with 2019-nCoV using real-time PCR techniques. Studies have shown that real-time PCR is one of the most accurate and effective methods to detect 2019-nCoV infection [3, 8]. Most patients with COVID-19 (11 of 13 cases) in the present study either traveled to Wuhan, Hubei, or were exposed to an individual who returned from Hubei in early January 2020. Two patients had no history of travel or contract during the time period. Most COVID-19 patients out of Wuhan or Hubei were cases involving second-generation human-to-human transmission that have been to Hubei or were exposed to one or more Hubei COVID-19 infection patients. The duration from initial onset to confirm infected 2019-nCoV was 3–11 days (mean 6.23 ± 2.42 days), and there were no statistical differences between mild and severe cases. There was potentially large first-generation human-to-human 2019-nCoV transmission in Wuhan, and Wuhan was also the major hub for the spread of the 2019-nCoV outbreak in other cities for approximately 1–2 weeks [9–12].

Results of our study suggest that there are no differences between age and sex with 2019-nCoV infection and the severity of COVID-19. However, patients with concomitant diseases, such as hypertension, diabetes and/or cerebral infarction, were significantly more likely to develop severe COVID-19-related disease. Another study suggested that elderly individuals experience faster disease progression and death [13].

A moderate degree of fever (38.0–39.0 °C), cough, fatigue, and gastrointestinal symptoms, such as anorexia, nausea, vomiting and diarrhea, were common symptoms among COVID-19 patients. A few patients experienced expectoration, dyspnea, and pharyngalgia, among others, which was similar to previous reports [3, 4]. However, in our study, two patients with mild COVID-19 infection did not exhibit fever, suggesting that fever was not the only clinical characteristic of COVID-19, which was different from COVID-19 patients in Wuhan [1, 14]. There is a difference between mild and severe cases in terms of degree of fever, days of persistent fever, cough, expectoration, fatigue, and other symptoms. Oxyhemoglobin saturation and oxygenation index are sensitive markers to evaluate the severity of COVID-19. Oxyhemoglobin saturation and oxygenation index decreased in the early period in cases with severe COVID-19; in contrast, most mild cases were normal [15].

CRP and SAA levels maybe effective biomedical indices for detecting COVID-19 infection. CRP and SAA levels increased rapidly during the early period of 2019-nCoV infection; moreover, indices of severe cases increased to higher levels than those with mild disease. However, white blood cells, neutrophils, lymphocytes, and monocytes demonstrated no significant changes during the early period of infection in both mild and severe cases. PCT levels also did not change.

Most COVID-19 patients in our study exhibited significant changes in chest CT imaging features. Chest CT is very important for the initial diagnosis of COVID-19 [16]. Chest CT may be helpful in diagnosing individuals with high clinical suspicion of 2019-nCoV infection but negative RT-PCR screening [17]. Typical CT imaging features include ground-glass densities, shadows or consolidations in both lungs [18, 19]. A recent study reported that in patients with mild COVID-19 infection, lung abnormalities on chest CT scan would recover approximately 10 days after the initial onset of symptoms [17]. In our study, most patients with mild COVID-19 (*n* = 5) chest CT abnormalities were gradually absorbed 5 days after confirmation of infection; however, most patients with severe disease experienced exacerbation of symptoms.

However, one mild COVID-19 case showed no significant chest CT image changes, which differed from Wuhan COVID-19 patients [20].

## Conclusions

In conclusion, results of the present study confirmed that most COVID-19 infections were second-generation human-to-human transmissions from other cities in China out of Wuhan, Hubei. The typical clinical characteristics of COVID-19 include a moderate degree of fever (37.3–38.0 °C), cough, fatigue, and gastrointestinal symptoms. Oxyhemoglobin saturation and oxygenation index changed significantly in severe cases. Serum levels of CRP and SAA were reliable biomarkers to evaluate the severity of COVID-19 infection. Chest CT was another effective method of detecting infection with 2019-nCoV. However, a few patients with mild COVID-19 disease without fever and CT imaging changes require the combination of additional screening methods.

## Data Availability

The datasets used and/or analyzed during the current study are available from the corresponding author on reasonable request.

## Abbreviations

2019-nCoV: Novel Coronavirus-2019
WHO: World Health Organization
SARS-CoV: Severe acute respiratory syndrome-related coronavirus
MERS-CoV: Middle East respiratory syndrome-related coronavirus
COVID-19: Coronavirus disease 2019
CRP: C-reactiveprotein
PCT: Procalcitonin
SAA: Serum amyloid A
CT: Computed tomography
